# Differentiation of Human Amniotic Mesenchymal Stem Cells into Human Anterior Cruciate Ligament Fibroblast Cells by In Vitro Coculture

**DOI:** 10.1155/2017/7360354

**Published:** 2017-09-20

**Authors:** Yuwan Li, Ziming Liu, Ying Jin, Xizhong Zhu, Shengmin Wang, Jibin Yang, Youliang Ren, Qiang Fu, Huazhang Xiong, Gang Zou, Yi Liu

**Affiliations:** ^1^The First Department of Orthopedics, The Affiliated Hospital of Zunyi Medical College, Zunyi, Guizhou 563000, China; ^2^The Department of Orthopedics, The First Affiliated Hospital of Chongqing Medical University, Chongqing 400016, China; ^3^Institute of Organ Transplantation, Sichuan Academy of Medical Sciences and Sichuan Provincial People's Hospital, School of Medicine, University of Electronic Science and Technology of China, Chengdu, Sichuan 610072, China

## Abstract

Anterior cruciate ligament injuries are common in humans, though cellular components of the knee have little regenerative or proliferation potential. This study investigated the differentiation of human amnion-derived mesenchymal stem cells (hAMSCs) into human anterior cruciate ligament fibroblasts (hACLFs) in vitro through induction with bFGF and TGF-*β*1 with coculture systems. Groups A and B comprised hAMSCs at the 3rd passage cultured with and without bFGF and TGF-*β*1, respectively; Groups C and D consisted of hAMSCs and hACLFs in monolayer coculture with and without bFGF and TGF-*β*1, respectively; Groups E and F were composed of hAMSCs and hACLFs in Transwell coculture with and without bFGF and TGF-*β*1, respectively. Cell morphology and proliferation were recorded. Protein expression and relative mRNA expression were evaluated in each group. Cell proliferation was significantly higher in the induced groups than in the noninduced groups. Protein expression increased over time with the highest expression observed in Group E. mRNA levels were significantly higher in Group E than in other groups. This study is the first to demonstrate the use of the Transwell coculture system for this purpose, and hAMSCs were successfully differentiated into hACLFs. Thus, hAMSCs may be a superior choice for hACLF differentiation via Transwell coculture.

## 1. Introduction

Knee injuries are common in sports, and the frequency of human anterior cruciate ligament (ACL) trauma or rupture has been increasing in recent years [1]. Due to the poor inherent healing capacity of the ACL, surgical reconstruction is considered indispensable. The objective of this well-accepted treatment intervention is to regain mechanical stability and reestablish knee function. Grafts used for reconstruction include autologous or allogeneic tendons and artificial ligaments, such as Ligament Advanced Reinforcement System [2, 3]. However, patients who undergo surgical or arthroscopic ACL reconstruction may experience osteoarthritis or degeneration after treatment; indeed, a study with a long-term follow-up period reported a prevalence of progression to osteoarthritis as high as 61% after 2–20 years [4]. Hence, the effects of anastomoses between the host tissue and selected graft are decisive for ACL reconstruction. Interestingly, a recent regenerative medicine study involving construction of a primary bioenhanced artificial ligament with multiple growth factors, including transforming growth factor (TGF)-*β*1, growth/differentiation factors 5 and 7, insulin-like growth factor 1, and basic fibroblast growth factor (bFGF)-2, showed that this approach can significantly decrease the prevalence of osteoarthritis in animals undergoing ACL reconstruction [5].

Nonetheless, tissue-engineered ligaments harboring seed cells have the greatest potential, as they are capable of synthesizing extracellular matrix (ECM) components, revascularization, and perivascular cell remodeling [6, 7]. There are many types of such seed cells, such as mature somatic cells or mesenchymal stem cells (MSCs) and especially bone marrow-derived stem cells (BMSCs), which have been widely applied due to their strong proliferation capacity and potential for differentiation into multiple other cell types [8–10]. However, the process of obtaining BMSCs is invasive, has risks for infection and bleeding, and may cause immune rejection after implantation in vivo, factors that may account for BMSCs not yet being successfully utilized in clinical reconstruction applications.

Previous studies have demonstrated derivation of human amnion-derived mesenchymal stem cells (hAMSCs) from the amniotic membrane on the placental surface. Due to their unique structure, noninvasive collection from an essentially unlimited source, utility in preparing grafts for soft tissue rehabilitation, and lack of ethical and moral controversy, hAMSCs have been widely applied in various fields [11–13]. Some research indicates that hAMSCs have strong potential, vitality, purity, and proliferation ability [14]. In addition, hAMSCs possess multidirectional differentiation capacity and exhibit low immunogenicity and stem cell properties. hAMSCs have also been extensively applied in treating bone and spinal injuries and neurological disorders, among other conditions [15–17]. Although they may display common characteristics of stem cells, different MSCs isolated from various sources may have different tissue-specific features and biological functions [18]. To date, multiple types of MSCs have been used as seed cells for constructing tissue-engineered cartilage, bone, and other soft tissues [19–21]. However, it remains to be determined whether hAMSCs, similar to BMSCs, possess the ability to differentiate into human anterior cruciate ligament fibroblasts (hACLFs). Our hypothesis for this study was that hAMSCs can better differentiate into hACLFs in coculture systems involving the application of bFGF and TGF-*β*1.

## 2. Materials and Methods

### 2.1. Groups for Differentiation of hAMSCs into hACLFs In Vitro

All experiments were repeated three times. The inducing factors used were 10 ng/ml bFGF and TGF-*β*1, and the differentiation time points were set at days 7, 14, and 21. Induced hAMSCs alone were used as Group A and noninduced hAMSCs alone as Group B; induced monolayer cocultured hAMSCs and hACLFs comprised Group C, and noninduced monolayer cocultured hAMSCs and hACLFs comprised Group D. Importantly, to inhibit proliferation of hACLFs, the cells were treated with 2 *μ*g/ml cytosine arabinoside for 24 h before monolayer cocultivation with hAMSCs. Group E consisted of induced Transwell cocultures of hAMSCs and hACLFs and Group F of noninduced Transwell cocultures of hAMSCs and hACLFs.

### 2.2. Cell Isolation and Culture

#### 2.2.1. Isolation and Culture of hAMSCs

This study was approved by the Research Ethics Committee of Zunyi Medical College Affiliated Hospital, and informed consent was provided by all patients before surgical retrieval and application. Placentas were obtained from the Obstetrics Department of the Affiliated Hospital of Zunyi Medical College. hAMSCs were isolated from 5 full-term puerperants, and informed consent was provided by the patients before delivery. The amniotic membrane was bluntly dissected from the placenta, and the samples were quickly transferred to sterile collection bottles and stored at 4°C in a laboratory facility. Using a laminar-flow bench, the amniotic membrane was placed in a sterile glass dish with phosphate-buffered saline (PBS) containing 1% penicillin and streptomycin and rinsed at least three times with PBS. The isolated tissue was minced with sterile scissors into pieces 1-2 mm^3^ in size, digested twice with 0.05% trypsin/0.01% ethylenediaminetetraacetic acid disodium salt (EDTA-2Na) for 30 minutes each, rinsed with PBS, and incubated for 1 to 2 hours with 0.75% collagenase type II in low-glucose Dulbecco's modified Eagle's medium (LG-DMEM) with 1% penicillin and streptomycin in water at 37°C, until the pieces were indistinct. hAMSCs were passed through a 300-mesh filter and collected into 50 ml centrifuge tubes. The cells were centrifuged at 1200 ×g for 5 minutes and then resuspended in LG-DMEM medium with 10% fetal bovine serum (FBS), 1.176 g NaHCO_3_, 1% penicillin and streptomycin, 1% L-glutamine, and nonessential amino acids and placed into T-75 flasks. The cells were incubated at 37°C with 5% humidified CO_2_. Every three days, the medium was refreshed after washing the cells three times with PBS. To passage the cells upon reaching 80–90% confluence, they were digested for 3 minutes with 0.125% trypsin/0.01% EDTA-2Na and then divided at a 1 : 2 ratio for subculture. Third-passage (P3) cells were used for subsequent experiments.

#### 2.2.2. Isolation and Culture of hACLFs

ACL tissues were isolated from 5 adult patients who underwent ACL reconstruction by arthroscopy after suffering ACL rupture. Basic information about these patients is shown in Table 1. Patients underwent general anesthesia after preoperative injection with antibiotics; a tourniquet was applied, and the systolic blood pressure was brought to above 120 mmHg, per the standard protocol for such patients. During the surgery, the membranous synovium was debrided using a notch above the ACL; then, a 10 mm scalpel was used to identify and collect the ACL stump. The sample was maintained in normal saline in a sterile centrifuge tube and delivered as quickly as possible to the laboratory facility for storage at 4°C. The patients recovered and underwent rehabilitation according to the standard postoperative protocol of our institution.

The human ACL tissues were transferred under sterile conditions to Petri dishes containing PBS and 1% penicillin and streptomycin. Connective tissues, including hematoma and vascular and adipose tissues, were scraped away as much as possible. The ACL tissue was minced with sterile scissors into pieces approximately 1 mm^3^ in size and rinsed with PBS three times; the pieces were placed in a T-25 flask with culture medium, resuspended in LG-DMEM-F12 with 10% FBS, 1.176 g of NaHCO_3_, 1% penicillin and streptomycin, 1% L-glutamine, and nonessential amino acids, and placed in T-25 flasks. The cells were incubated at 37°C with 5% humidified CO_2_. The medium was refreshed every four to five days after washing the cells three times with PBS. To passage the cells, they were digested for 5 minutes with 0.125% trypsin/0.01% EDTA-2Na upon reaching 80–90% confluence and then divided at a 1 : 2 ratio for subculture. P3 or P4 cells were used for subsequent experiments. hACLFs were treated with 2 *μ*g/ml cytosine arabinoside (Arab) for 24 hours before use in the monolayer coculture system.

### 2.3. Phenotypic Identification and Multidirectional Differentiation Potential of hAMSCs

P3 hAMSCs were subjected to cell marker detection by flow cytometry. The differentiation potential of hAMSCs into osteoblast-like cells, chondrocytes, and lipocytes was observed by alizarin red, toluidine blue, and oil red O staining.

The cell density was adjusted to 2 × 10^6^/ml, and 100 *μ*l of the cell suspension was transferred to a tube for flow cytometry. The cells were incubated with fluorescein isothiocyanate- (FITC-) conjugated anti-CD90, phycoerythrin- (PE-) conjugated anti-CD44, peridinin chlorophyll protein-conjugated anti-CD105, and allophycocyanin-conjugated anti-CD73. The cells were incubated with the negative control antibodies, including PE-conjugated anti-CD34, anti-CD19, anti-CD45, anti-CD11b, and anti-HLA-DR, for 30 minutes in the dark, washed by addition of 2 ml of flow buffer, and centrifuged at 1200 ×g for 5 minutes. The liquid supernatant was removed, and the cells were resuspended in 250 *μ*l of flow buffer. Flow cytometry in conjunction with Coulter Epics XL software (Beckman Corporation) was used to analyze hAMSC surface marker expression.

hAMSC expression of CK-19 and vimentin was detected by immunofluorescence. Nonspecific goat IgGs were added to isotype controls to eliminate nonspecific staining in the two groups. P3 hAMSCs on cover slips in 6-well cell culture plates were fixed with 4% paraformaldehyde, and PBS-Tween 20 (PBST) was used to wash the cover slips. The cell membrane permeability was then increased with 0.1% polyethylene glycol octylphenol ether (Triton X-100) for 10 minutes, and the cells were blocked with Lowenthal serum for 30 minutes. The cells were incubated with purified primary antibodies overnight or for 12 hours and then with secondary FITC-labeled antibodies for one hour. Cell nuclei were counterstained with 2-(4-amidinophenyl)-1H-indole-6-carboxamidine at room temperature for 5 minutes. The cover slips were slowly mounted onto slides with 90% glycerol and then observed by inverted fluorescence and phase-contrast microscopy.

The potential of hAMSCs to differentiate into osteoblast-like cells, chondrocytes, and lipocytes was observed after the cells reached 80–90% confluence and were cultured in osteogenic, chondrogenic, or adipogenic medium.

### 2.4. Determination of hAMSC Proliferation by Cell Counting Kit- (CCK-) 8 Assays

P3 cells in each group were grown to 85% confluence, and cell suspensions at 10^5^ cells/ml were prepared; 100 *μ*l of each cell suspension was added to a standard 96-well plate. The cells were continuously cultured for 7 days, and each group included 5 replicate wells. Viability was evaluated in all of the wells by using CCK-8 assays. Absorbance at 450 nm was detected using a microplate reader (A5082-TECAN), growth curves were drawn, and the cell proliferation activity of each group was observed.

### 2.5. Immunofluorescence Staining of hAMSCs

Expression of collagen types I and III, fibronectin, and tenascin-C in each group was evaluated by immunofluorescent staining. Cells of each group were seeded onto sterile cover slips in Corning 12-well plates at a density of 10^4^ cells/ml in LG-DMEM-F12 medium with 10% FBS with or without bFGF and TGF-*β*1. The cells were incubated at 37°C with 5% humidified CO_2_ for 7, 14, and 21 days. After incubation, the cells in each group were washed 3 times with ice-cold PBS for 10 minutes each. The cells were then fixed with 4% paraformaldehyde at 37°C for 15 minutes in a thermostatic water bath, washed 3 times with PBS for 10 minutes each, and permeabilized using 0.3% Triton X-100 for 30 minutes at 37°C. Cells in each group were blocked with goat serum for 40 minutes after permeabilization. The cells were then incubated overnight with the primary antibodies rabbit monoclonal anti-human collagen types I (ab34710; Abcam) and III (ab7778; Abcam) and mouse monoclonal anti-human fibronectin (ab6328; Abcam) and anti-tenascin-C (ab86182; Abcam), followed by incubation with the corresponding fluorophore-conjugated antibodies for 60 minutes. The cells were washed 3 times with 0.1% PBST for 10 minutes each, and the cover slips were carefully removed and then mounted on slides with glycerol. The same protocol was performed in the negative control groups except that the primary antibodies were omitted. The mounted slides were observed by inverted fluorescence microscopy (Oly 3800; Olympus), and the images were analyzed using an Olympus auxiliary system. The immunofluorescent staining results were quantified and recorded using Image Pro Plus software.

P3 hAMSCs and hACLFs on cover slips were examined using the staining protocol described above, except that the cells were or were not incubated in the permeabilization solution.

### 2.6. Picrosirius Red Staining Analysis of Collagen Expression in Each Group

On days 7, 14, and 21, cells from each group were fixed with 4% paraformaldehyde at 37°C for 15 minutes after washing 3 times with PBS. After the cells were washed another 3 times, the picrosirius red staining results were observed by inverted phase-contrast microscopy according to the manufacturer's instructions. The staining results were quantified and recorded with Image Pro Plus software.

### 2.7. Real-Time Quantitative Reverse Transcription Polymerase Chain Reaction (qRT-PCR) Analysis of Relative Gene Expression Levels

Cells in each group were cultured in LG-DMEM-F12 medium with or without bFGF and TGF-*β*1. The control group consisted of hAMSCs incubated in LG-DMEM-F12 medium. After cDNA was obtained from total RNA extracted from cells in each group on days 7, 14, and 21, gene expression of collagen types I and III, fibronectin, tenascin-C, matrix metalloproteinase (MMP)-2, lysyl oxidase- (LOX-) 1 was detected by real-time qRT-PCR. The amplification conditions included predenaturation at 95°C for 30 seconds, denaturation for 5 s, and annealing at 60°C for 30 seconds. Human glyceraldehyde 3-phosphate dehydrogenase (GAPDH) was used as the internal reference. The PCR primers are provided in Table 2. The relative expression levels of genes among the groups were analyzed using the 2^ΔΔCT^ method.

### 2.8. Western Blot Analysis of Collagen I and III Expression

On days 7, 14, and 21, cells from each group were washed with PBS and digested in 0.125% trypsin at 37°C for 5 minutes before collection and centrifugation. The reaction was stopped with LG-DMEM solution containing 10% FBS. Total cellular proteins were extracted using radioimmunoprecipitation assays according to the manufacturer's protocol. Protein concentrations were assessed using a bicinchoninic acid protein assay kit (23325; Thermo). The appropriate quantity of sodium dodecyl sulfate polyacrylamide gel electrophoresis (SDS-PAGE) loading buffer was added to the proteins, followed by boiling for 5 minutes; aliquots were stored at −80°C until western blot analysis. Protein expression of collagen I and collagen III was measured in each sample using human GAPDH as a control for equal protein loading.

Total proteins were denatured and separated using precast 5–12% SDS-PAGE gels with Tris-glycine buffer. PageRuler Plus Prestained Protein Ladder (26619; Thermo Scientific) was used to evaluate the bands based on molecular weights ranging from 10 to 250 kDa. The proteins in the gels were carefully transferred onto polyvinylidene difluoride (PVDF) membranes under dark conditions. The membranes were blocked with 5% evaporated milk for 2 hours at room temperature and incubated overnight at room temperature with polyclonal rabbit anti-human collagen I (ab34710; Abcam) or III (ab7778; Abcam). After the membranes were washed with Tris-buffered saline/Tween 20 (TBST) 3 times for 10 minutes, the membranes were probed with a fluorescently labeled goat polyclonal anti-rabbit secondary antibody (LI-COR Odyssey Company). The fluorescent signal was captured using a LI-COR Odyssey Infrared Imaging System. In addition, the membranes were incubated with a monoclonal mouse anti-human GAPDH (60004-1; Proteintech) antibody used as a loading control for collagen type I or III. The same signal system and procedures for human GAPDH loading controls were applied as described above. Relative band intensity was measured using Image J analysis software.

### 2.9. Statistical Analyses

Differences among groups were assessed using a three-way analysis, and the data are reported as the mean ± standard error of the mean. Statistical analyses were performed using the software package SPSS 14.0, and Fisher Exact tests and Student–Newman–Keuls *q* tests were conducted to identify significant differences among groups. Statistical significance was set at an *α* level of 0.05 for all post hoc comparisons.

## 3. Results

### 3.1. Culture and Morphology of hAMSCs and hACLFs

**Figure 1:**
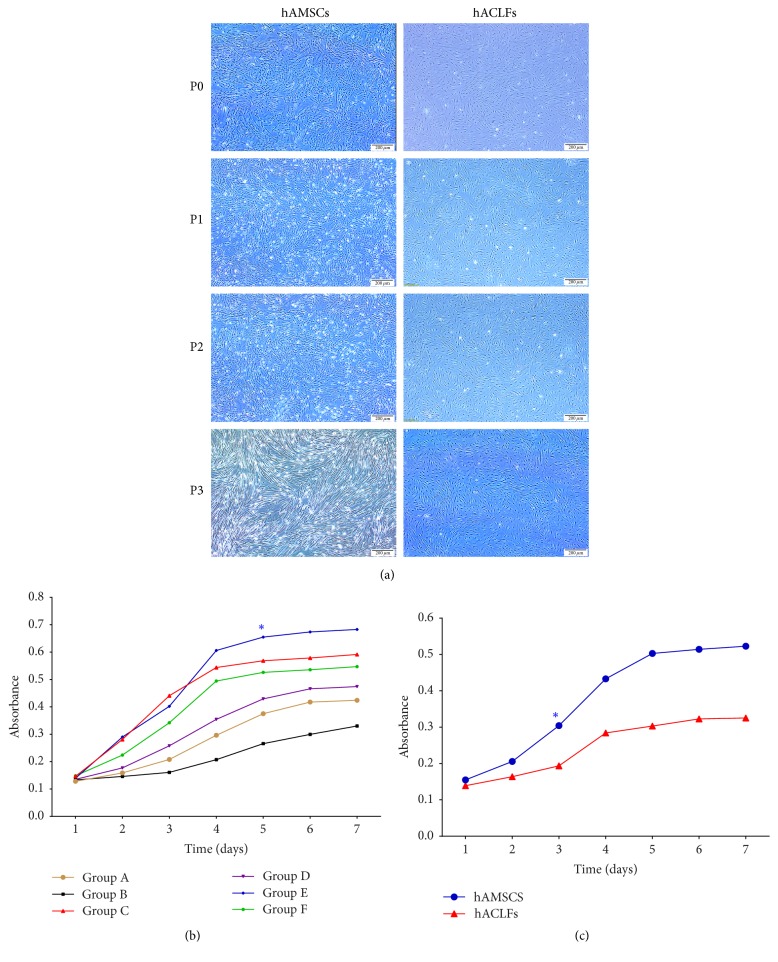
Morphology of cultured hAMSCs and hACLFs; the proliferative curve of cells in each group, as determined by CCK-8. Primary cultures of hAMSCs and hACLFs were cultured to the third passage (a) (original magnification 40x). Proliferation of hAMSCs in each group, as determined by the CCK-8 method showed that cells in Group E multiplied faster than those in Group C and other groups and that cells in Group B grew most slowly (b). The proliferative curves of hAMSCs and hACLFs based on the CCK-8 method showed a doubling time for hAMSCs and hACLFs as high as 32.5 h and 49.8 h, respectively; the proliferation rate of hAMSCs was significantly higher than that of hACLFs (c) (^*∗*^*P* < 0.05, scale bar in (a) = 200 *μ*m).

The morphology of the hAMSCs and hACLFs was observed after 24 hours in primary culture. P1, P2, and P3 hAMSCs showed a uniform spindle-shaped exterior with spiral and radial-like growth, and increasing polarization was observed with each passage. During primary isolation and culture, both cell types were adherent and presented a spindle-shaped, fibroblast-like appearance. The hAMSCs reached approximately 80–90% confluence after 5 days, whereas the hACLFs usually required approximately 10 days. The hACLFs showed a radial growth arrangement around the center of adherence. After approximately 15 days in primary culture, many hACLFs had migrated from the edge of the tissue block and connected with each other. The hAMSCs and hACLFs usually required approximately 7 and 21 days, respectively, to cover the culture flask. P1, P2, and P3 hACLFs exhibited a long, thin spindle shape, and partially vortex-like cell colonization was observed. hACLF nuclei presented an oval shape, and two nucleoli were occasionally observed (Figure 1(a)).

### 3.2. Proliferation of Cells in Each Group, as Determined by CCK-8

The proliferative curve of cells in each group showed an “S” pattern. After 1 day of latency, the cultured cells in each group went through a logarithmic growth phase for 4 to 5 days, after which the multiplication rate was maintained (Figure 1(b)).

The growth curves for hAMSCs and hACLFs showed distinct differences in proliferation. The hAMSCs grew faster than the hACLFs during the latency period and the logarithmic growth phase. During the plateau phase, hAMSC proliferation continued to increase, though that of hACLFs remained stable. The doubling times of the hAMSCs and hACLFs were 32.5 h and 49.8 h, respectively (Figure 1(c)).

### 3.3. Phenotypic Properties of hAMSCs

#### 3.3.1. Flow Cytometric Analysis and Multidirectional Differentiation Potential of hAMSCs

**Figure 2:**
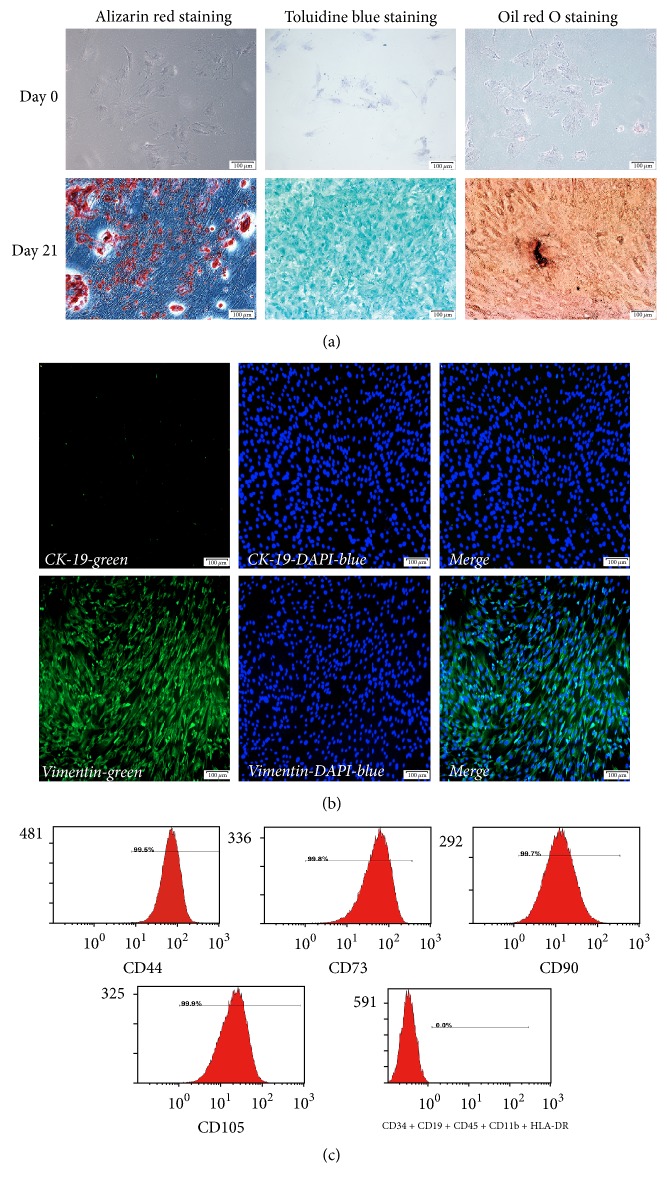
Phenotypic properties of hAMSCs and multidirectional differentiation potential of hAMSCs. Alizarin red staining of hAMSCs cultured in osteogenic medium for 0 and 21 days. Toluidine blue staining of hAMSCs cultured in chondrogenic medium for 0 and 21 days. Oil red staining of hAMSCs cultured in adipogenic medium for 0 and 21 days (a). hAMSCs in the 3rd passage barely expressed CK-19. hAMSCs were positive for vimentin expression. CK-19 and vimentin were stained green by fluorescein isothiocyanate (FITC), and cell nuclei were stained blue by 4′,6-diamidino-2-phenylindole (DAPI) (b). According to flow cytometric analysis, hAMSCs were positive for expression of CD90, CD105, CD73, and CD44 and negative for expression of CD34, CD19, CD45, CD11b, and HLA-DR (c). Scale bars in (a-b) = 100 *μ*m.

According to flow cytometry, P3 hAMSCs highly expressed mesenchymal markers CD90, CD73, CD105, and CD44 and weakly expressed hematopoietic markers CD34, CD19, CD45, CD11b, and HLA-DR (Figure 2(c)). In addition, the potential for multidirectional differentiation into osteoblast-like cells, chondrocytes, and lipocytes was demonstrated by the results of various staining procedures (Figure 2(a)).

#### 3.3.2. Osteoblast-Like Cells

These cells gathered and became compact after induction for 7 to 10 days, and their morphology became more deltoid and polygonal in shape, as opposed to the original spindle-like appearance. With increasing induction time, osteogenic nodules grew larger and became more symmetrically distributed. The differential potential of hAMSCs into osteoblast-like cells was determined by alizarin red staining after 0 and 21 days. As early markers of bone differentiation, red mineralized matrix and nodules were observed after alizarin red staining (Figure 2(a)).

#### 3.3.3. Chondrogenesis

The spindle shape of these cells gradually became shorter or disappeared, and the karyoplasmic ratio decreased after induction for 7 days. This phenomenon was more obvious after 21 days of induction. As glycosaminoglycans are secreted by chondrocytes, the ECM became blue when the cells were stained with toluidine blue (Figure 2(a)).

#### 3.3.4. Adipogenesis

These cells showed a disordered arrangement, and the morphology became circular; lipid droplets were observed after incubation in adipogenic culture medium for 7 days. Highly refractive lipid droplets were observed after induction for 21 days, and the graininess of the lipid droplets in the cytoplasm appeared red under high-power microscopy after oil red O staining (Figure 2(a)).

#### 3.3.5. CK-19 and Vimentin Expression in hAMSCs

CK-19 is a specific marker of human amniotic epithelial cells (hAECs), and vimentin is a marker of hAMSCs. Small quantities of hAECs were present during the isolation of hAMSCs from human amnion samples. However, with each passage, the hAECs appeared to gradually pass through the epithelial to mesenchymal transition. Based on immunofluorescence, P3 hAMSCs were positive for vimentin expression and negative for CK-19 expression (Figure 2(b)).

### 3.4. Immunofluorescence Analysis of hAMSC Ligament-Specific Protein Expression

**Figure 3:**
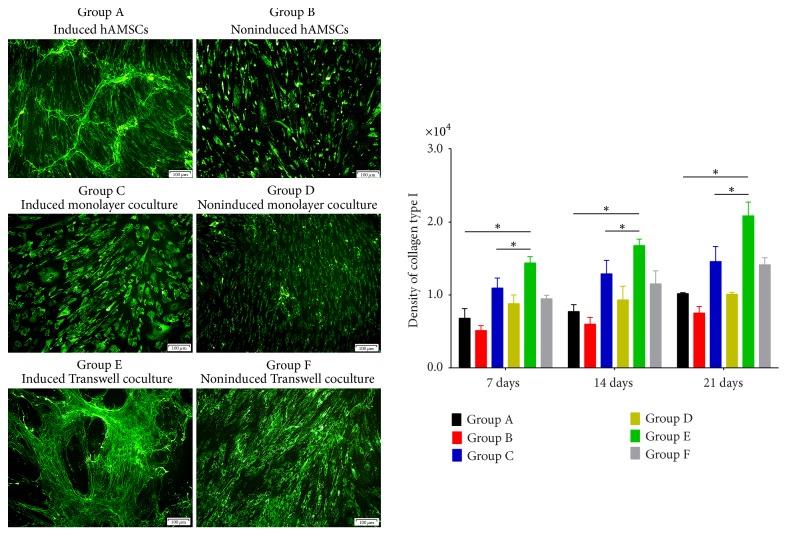
Expression of collagen type I on day 21 and its quantification in each group based on immunofluorescence staining. Expression of collagen type I over time in all groups and intracellular secretion. Expression of collagen type I in Group C increased on days 7, 14, and 21. Expression of collagen type I was greater in Group C than in Group D at the same time points. Expression of collagen type I was greater in the coculture groups than in Group A and on days 7, 14, and 21, and it was greater in Group E than in other groups (original magnification ×100, scale bar = 100 *μ*m) (^*∗*^*P* < 0.05).

**Figure 4:**
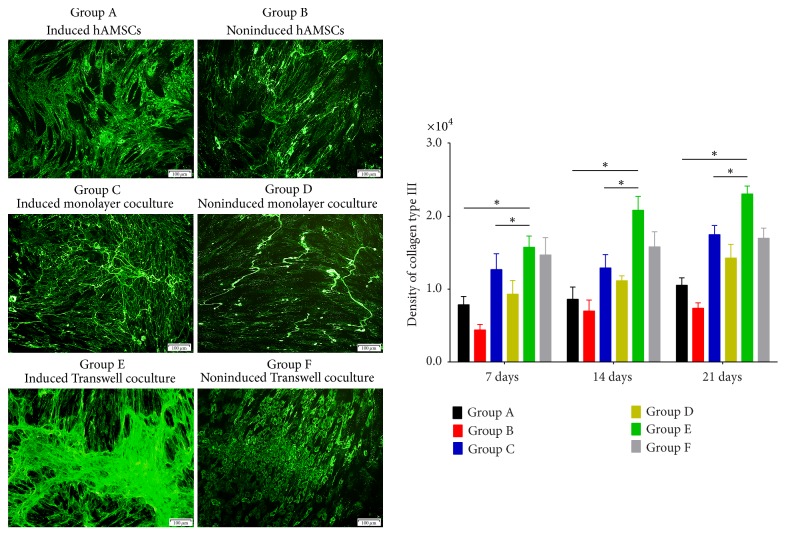
Expression of collagen type III on day 21 and its quantification in each group based on immunofluorescence staining. Collagen type III was secreted intracellularly and showed significantly increased expression over time in Group C, in which a small amount of fusion began on day 7; on days 14 and 21, larger shapes formed by collagen type III protein were observed in Group C compared to in Groups A, B, and D on days 7, 14, and 21. Group E showed strong expression of collagen type III, with the highest expression on day 21, and higher expression than in other groups at the same time points (original magnification ×100, scale bar = 100 *μ*m) (^*∗*^*P* < 0.05).

**Figure 5:**
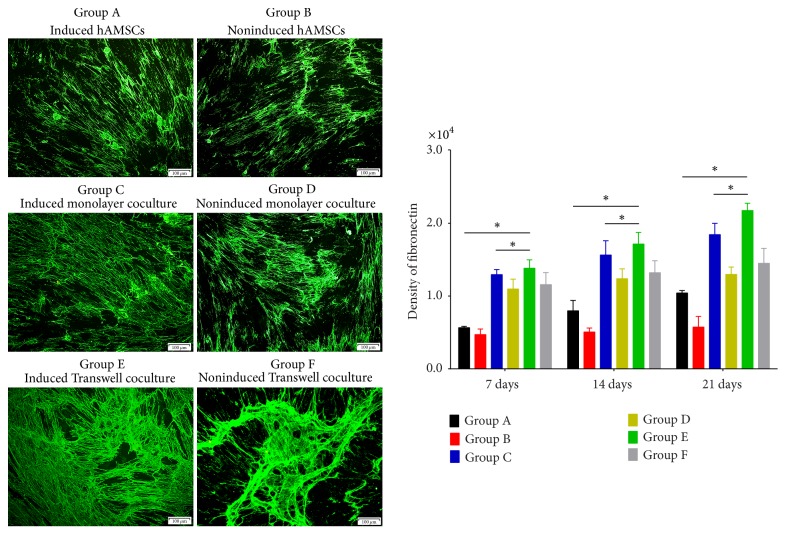
Expression of fibronectin on day 21 and its quantification in each group based on immunofluorescence staining. Fibronectin was secreted both intracellularly and extracellularly. Compared with other groups at the same time points, Group E showed a higher level of fibronectin expression and exhibited fusion. Group C showed high fibronectin expression, and fusion was observed on days 7, 14, and 21. Expression of this protein was significantly greater in Group C than in Groups A, B, and D (original magnification ×100, scale bar = 100 *μ*m) (^*∗*^*P* < 0.05).

**Figure 6:**
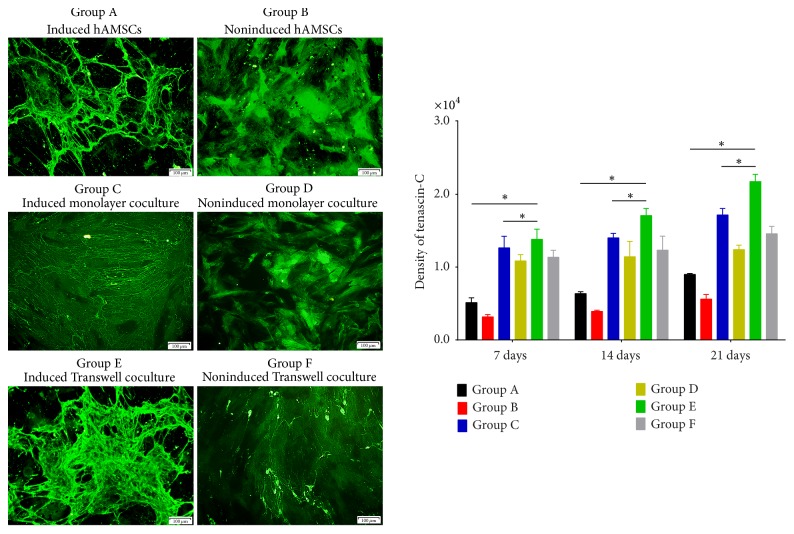
Expression of tenascin-C on day 21 and its quantification in each group based on immunofluorescence staining. Tenascin-C was mainly secreted extracellularly and appeared as a large, misty cloud shape; this protein was not observed in nuclei. Group E showed a significant increase in tenascin-C expression that increased over time, and tenascin-C expression was highest in Group E on days 7, 14, and 21. Group F showed limited tenascin-C expression and maintained a fixed level of protein secretion over time. On days 7, 14, and 21, Group C showed strong expression of tenascin-C compared with Groups A, B, and D (original magnification ×100, scale bar = 100 *μ*m) (^*∗*^*P* < 0.05).

Confocal fluorescence microscopy of the immunofluorescence results illustrated the distributions of differentiated collagen I and III, fibronectin, and tenascin-C as well as protein secretion among the groups (Figures 3–6). The distributions of collagen I and III, fibronectin, and tenascin-C demonstrated extracellular secretion.

Collagen I and III staining was observed around the cell nucleus, and more spreading, web-like agglomerates sprouting from the nucleus were observed over time. Fusion began in Groups C and E on days 14 and 21, and expression in Group E was highest on day 21 (Figures 3-4). However, expression of collagen types I and III in the noninduced groups increased only slightly over time.

The results of fibronectin immunofluorescence showed the presence of this protein around the cell nucleus, with strong expression in Groups C and E; fusion was observed on day 21, and Group E expressed the highest level of fibronectin (Figure 5). Expression of fibronectin in the non-bFGF- and TGF-*β*1-treated groups was relatively invariant.

Tenascin-C immunofluorescence demonstrated development of a dispersed, web-like protein formation around the cell nucleus, with high fusion density on days 14 and 21. Fusion was brightly and clearly observed in the bFGF- and TGF-*β*1-treated groups, with Group E expressing the highest level of tenascin-C (Figure 6). In contrast, expression of tenascin-C in the noninduced groups increased only slightly over time.

### 3.5. Picrosirius Red Staining Analysis

**Figure 7:**
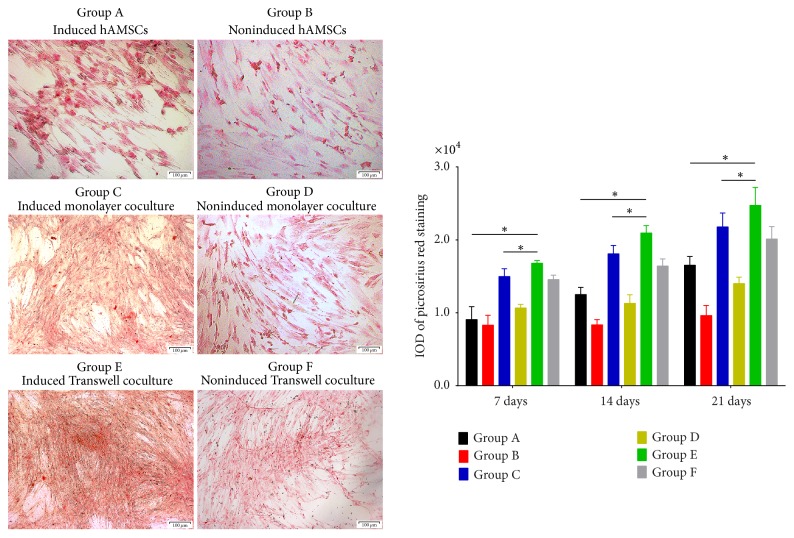
Collagen expression on day 21 and its quantification in each group based on picrosirius red staining. The results of picrosirius red staining showed total collagen secretion in each group; collagen was mainly produced intracellularly, and some secretion into the cytoplasm was observed. Group E showed a significantly increased level on day 21. Group F showed a lesser degree of collagen secretion than Group E. Total collagen expression in Group C was significantly increased on days 7, 14, and 21 and was higher than that in Groups A and B. Group A showed higher collagen secretion on days 7, 14, and 21 compared to Group B, which showed limited collagen production and fixed expression at the same time points (original magnification ×100, scale bar = 100 *μ*m) (^*∗*^*P* < 0.05).

The picrosirius red staining results, which were observed by inverted phase-contrast microscopy, showed the secretion and distribution of collagen. Picrosirius red staining revealed significantly increased collagen secretion around the cell nucleus in the induced groups on day 21, in comparison with days 14 and 7. Group E expressed the highest levels of collagen, whereas the noninduced groups generally showed little to no increase in staining intensity on days 14 and 21 compared with day 7 (Figure 7).

### 3.6. Expression of Ligament-Related Genes

**Figure 8:**
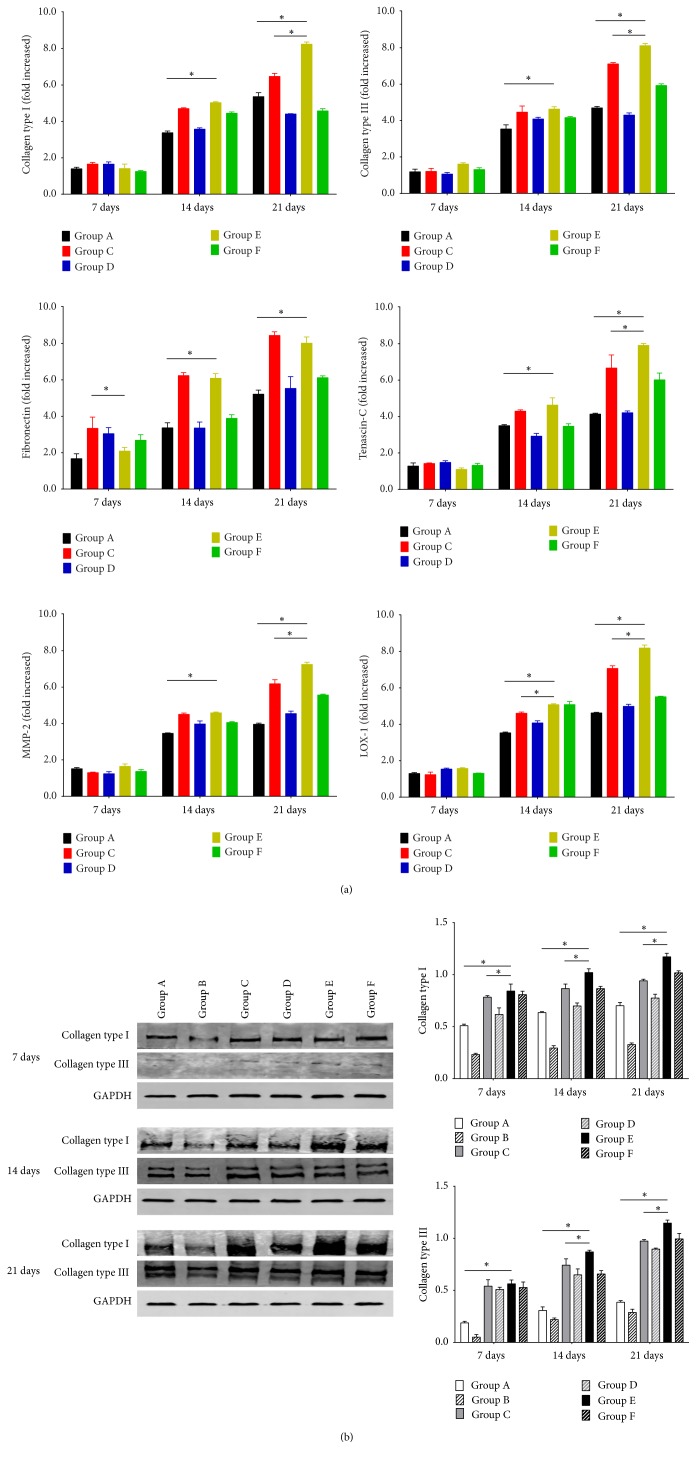
mRNA levels of collagen types I and III, fibronectin, tenascin-C, MMP-2, and LOX-1 and protein expression levels of collagen types I and III in hAMSCs were evaluated in each group. Group B served as the control group. Compared with the noninduced groups, the induced groups showed significantly increased expression on days 7, 14, and 21. Among all groups, Group E showed the greatest increase in expression on day 21. Group E also showed the highest level of collagen type III mRNA expression. Fibronectin expression in Group E differed significantly from the other groups on day 21. Group E also showed higher expression of MMP-2 on day 21 compared with the control group. mRNA expression of LOX-1 was similar in all groups, but its level in Group E was higher than that in the other groups on day 21 (a) (^*∗*^*P* < 0.05). Expression levels of collagen types I and III in coculture systems with and without induction on days 7, 14, and 21, as determined by western blotting. Group E expressed the highest levels of collagen types I and III (b) (^*∗*^*P* < 0.05).

Compared with noninduced hAMSCs, that is, the control group, expression of the 7 investigated ligament-related genes in each group had increased less than 5-fold by days 7 and 14. With no exceptions, every gene in each group showed significant upregulation on days 14 and 21 compared with day 7. On day 21, significant increases in collagen type I mRNA levels, 6.4- and 8.2-fold, were observed for the induced monolayer coculture and Transwell coculture groups, respectively, compared with the control group; on day 21, all coculture groups showed increases in collagen type III expression greater than 4-fold. mRNA expression of fibronectin was significantly increased in the induced monolayer coculture and Transwell coculture groups, 8.4- and 8.0-fold, respectively. Compared with the control group, expression of tenascin-C in the induced monolayer coculture and Transwell coculture groups increased 7.9- and 6.6-fold, respectively, on day 21. In all coculture groups, expression levels of MMP-2 and LOX-1 on day 21 were greater than in the control group, more than 3.9-fold and 4.1-fold, respectively (Figure 8(a)).

### 3.7. Western Blot Analysis

Western blot results for collagen types I and III revealed variations in expression among the different culture system and induction conditions. Collagen type I protein expression in Groups C and E was significantly higher on day 21 than on days 14 and 7, and Group E showed the highest expression. A similar pattern was observed for collagen type III protein in the induced monolayer and Transwell groups, with expression on day 21 being the highest in Group E. Although increased expression of these two proteins was found in each group, the non-bFGF- and TGF-*β*1-treated groups exhibited little or no increase, and the levels of these two proteins were higher in the noninduced Transwell groups than in the other noninduced groups at the same time points (Figure 8(b)).

## 4. Discussion

ACL injury or rupture occurs frequently. Despite some reports that the ACL has some self-healing ability with suture repair after injury, repair of ruptured ACL tissue usually requires reconstructive surgery [22–24]. In recent years, many studies have demonstrated that biological treatment modalities contribute to ACL healing [25]. Tissue engineering techniques have three essential elements, seed cells, growth factors, and scaffolds, providing new treatment options for ligament injuries [26]. Many types of MSCs have been discovered, including BMSCs, adipose-derived MSCs, chorionic-derived MSCs, and peripheral blood-derived MSCs. As representative MSCs, BMSCs are reported to have the potential for differentiation into osteoblasts, chondrocytes, tenocytes, myocardiocytes, and hepatocytes [27–31]. However, the process of extracting BMSCs is invasive, and there is a high risk of infection, hemorrhage, or immunological rejection. There are several recent reports of hAMSCs as newly discovered MSCs [32, 33]. hAMSCs are abundantly available from placentas after birth; in addition to other exceptional benefits, their extraction causes no trauma; there are no ethical or moral limitations.

In this study, we successfully used enzymatic digestion to isolate a large quantity of hAMSCs with high activity. CCK-8 results demonstrated that hAMSC proliferation peaked on days 4-5, with overall excellent proliferation. Flow cytometric analysis showed that these hAMSCs expressed CD90, CD44, CD73, and CD105 but did not express CD34, CD19, CD45, CD11b, and HLA-DR. Parolini et al. [34] reported that hAMSCs express low levels of the immunological factor CD14, the main histocompatibility type I antigens HLA-A, HLA-B, and HLA-C, and costimulatory molecules such as CD80, CD83, CD86, and CD40L. In our study, hAMSCs showed low expression of CD45 and HLA-DR, demonstrating that these cells have low immunogenicity and would not cause a lymphocyte proliferation reaction. Hence, hAMSCs can be a cellular resource for repairing tissue or organ damage without inducing immune rejection.

As hACLFs are isolated from mesoderm-derived connective tissue, we hypothesized that hAMSCs have the ability to differentiate into hACLFs via tissue engineering. Coculture systems can induce cell differentiation, maintain cellular activity and function, regulate proliferation, and improve metabolite production [35]. There are many types of coculture systems, including mixed culture, monolayer, Transwell, and three-dimensional coculture systems. Leyh et al. [36] implemented a coculture system with BMSCs and osteoarthritic cartilage in various proportions and found that chondrocyte proliferation and ECM synthesis were positively correlated with the proportion of BMSCs. Therefore, coculturing can markedly improve chondrocyte multiplication without the use of growth factors, thereby reducing the number of required cartilage cells. Accordingly, coculturing MSCs and hACLFs may be a practical strategy for the optimization and expansion of seed cell sources.

In this study, by using monolayer and Transwell coculture systems, we discovered that expression of ligament-related genes and proteins increased during the induction process. After evaluating immunofluorescent staining of proteins by confocal fluorescence microscopy and a picrosirius red assay, we found collagen types I and III to be more highly expressed in Group E than in the other groups at the same time points and that the levels of protein expression in the induced groups were higher than those in the noninduced groups. Based on the fusion phenomenon observed in the immunofluorescence assay, the bioactivity of hAMSCs in some coculture groups increased over time. This might be a result of the hACLFs producing cytokines that regulate and control hAMSC proliferation and secretion activities. Via the connection of the upper and lower chambers with a 0.4 *μ*m Transwell membrane, the Transwell coculture system can mimic the in vitro environment of hACLFs that induces differentiation in hAMSCs.

Accumulating evidence has shown that growth factors not only enhance revascularization and ligament-oriented differentiation and formation but also improve collagen remolding in the ligament restoration process. bFGF belongs to the heparin-binding growth factor family. Previous studies [37, 38] have reported that bFGF and TGF can be used to improve the bioactivity of osteoblasts and promote the aggregation of fibroblast and inflammatory cells, further enhancing the formation of blood vessels and granulation tissue.

Collagen types I and III are bioactive substances that are widely present in cartilage, tendons, and other connective tissue; indeed, these fibers constitute the framework of tendons [39, 40]. TGF belongs to the TGF superfamily, which regulates cell growth and differentiation and modulates tissue inflammation and repair [41–43]. TGF plays a role in activating neutrophils, monocytes, fibroblasts, and T cells and rebuilds ECM and granulation tissue via angiogenesis during inflammation [44]. Our results showed upregulated mRNA expression of collagen types I and III, fibronectin, tenascin-C, LOX-1, and MMP-2. Because LOX-1 and MMP-2 are expressed by fibroblasts, adventitial cells, and fibrocartilage cells and activate the contractile function of cells during connective tissue injury [45], our results suggest that the hACLFs in our study underwent a self-recovery process after isolation.

In this study, we applied single induction, monolayer, and Transwell coculture systems with bFGF and TGF-*β*1 to improve the bioactivity of hAMSCs in the hACLF differentiation process. The principal results of this study support our hypothesis. The levels of collagen I and collagen III, fibronectin, and tenascin-C were detected, and we found that hAMSC differentiation into hACLFs was most successful under the induced Transwell coculture conditions. Our results also showed that bFGF and TGF-*β*1 enhance cell proliferation and secretion and upregulate mRNA expression of genes and proteins associated with ligament repair. Previous studies have reported that strengthening the biological healing of the human ACL is an effective strategy for improving ACL repair and that seed cells and growth factors play important roles in constructing tissue-engineered ligaments [46–48]. The findings of our study demonstrate that applying bFGF and TGF-*β*1 in a Transwell coculture system can improve the bioactivity of hAMSCs after differentiation. Similar differentiation experiments in previous studies mainly used animal-derived amniotic MSCs. In contrast, we isolated amniotic MSCs and ACL samples from humans to optimize the similarity of the in vitro seed cell conditions to the conditions in humans.

This study had some limitations. First, the timeframe for in vitro differentiation was short. However, this factor can be modified if desired. This experiment used only 3 time points to analyze the aspects of differentiation; although bFGF and TGF-*β*1 enhanced differentiation, a longer study should be conducted to observe whether this effect is maintained over time. Second, we identified the minimum criterion for defining MSCs, though uniformly standard criteria for hAMSCs are still lacking. Nonetheless, the human ACL is a hard connective tissue, and we observed expression of ligament-related genes and proteins. Third, our interpretation of the levels of mRNA and protein expression in the monolayer cocultures was limited due to the similar morphology of hAMSCs and hACLFs and their combination in coculture. Fourth, we determined that hAMSCs could differentiate into hACLFs in vitro, but we did not evaluate their migration efficiency, such as the extent and rate of differentiation. Future studies should focus more on fully evaluating the roles of bFGF and TGF-*β*1 in the differentiation process.

## 5. Conclusion

This study found that when compared with other induction methods the Transwell coculture system can better enhance the differentiation of hAMSCs into hACLFs in vitro. Our results indicate that this approach may be useful in the future for increasing the quality and proliferation of seed cells in constructing tissue-engineered ligaments. Due to their noninvasive collection from an essentially unlimited source, their utility in preparing grafts for soft tissue rehabilitation, and their lack of ethical and moral controversy, hAMSCs may be widely applied in tissue-engineered ligament reconstruction in the future.

## Figures and Tables

**Table 1:** Clinical history of the ACL rupture patients providing research materials.

Parameter	Mean ± Std Dev
Age (year)	22.8 ± 2.490
Sex	5 males
Time from injury (days)	2.4 ± 1.402
Time from surgery (days)	5.2 ± 1.303

**Table 2:** Primer sequences for genes associated with hACLFs.

Target	Primer sequence (5′ → 3′)	Product length (bp)
COL1-A1	Forward: TCCGACCTCTCTCCTCTGAA Reverse: TGCTTTGTGCTTTGGGAAGT	121
COL3-A1	Forward: TCAGGGTGTCAAGGGTGAA Reverse: CAGGGTTTCCATCTCTTCCA	131
Fibronectin	Forward: ATCACCCTCACCAACCTCAC Reverse: TCCCTCGGAACATCAGAAAC	122
Tenascin-C	Forward: GGCAGGTGTCTTTCTTGCTT Reverse: ACTGGCTGGTTCTCTTCTGG	120
MMP-2	Forward: TATGGCTTCTGCCCTGAGAC Reverse: CACACCACATCTTTCCGTCA	142
LOX-1	Forward: TTACCCAGCCGACCAAGATA Reverse: CCTTCAGCCACTCTCCTCTG	122
GAPDH	Forward: CATGTTTGTGATGGGCGTGAA Reverse: AATGCCGAAGTGGTCGTGGAT	70
